# Phytophotodermatose aux algues marines de localisation inhabituelle

**DOI:** 10.11604/pamj.2013.15.58.2951

**Published:** 2013-06-20

**Authors:** Karima Chakir, Hakima Benchikh

**Affiliations:** 1 Service de dermatologie et vénérologie, CHU, Ibn Rochd, Maroc

**Keywords:** Phytophotodermatoses, produits photosensibilisants, placard érythémateux, antihistaminique, Phytophotodermatoses, photosensitizing products, erythematosus spot, antihistaminic

## Image en médicine

Les Phytophotodermatoses sont des réactions phototoxiques provoquées par le contact avec des plantes contenant des produits photosensibilisants (furocoumarines, psoralènes et isopsoralènes). Ils sont dus à l'action combinée du contact avec la plante, l'exposition aux UVA et d'un milieu humide favorisant la diffusion sur la peau des molécules photosensibilisantes. Nous rapportons une observation pédiatrique d'une fillette âgée de 5 ans sans antécédent particulier qui suite à une baignade dans la mer elle a eu un contact direct des fesses avec les algues en position assise suivie d'une sensation de cuisson, de brûlure, quelques heures après apparition d'une éruption cutanée aigue prurigineuse, nausées et vomissements. L'examen général montrait une enfant apyrétique, de bonnes constantes hémodynamiques. L'examen dermatologique retrouvait un placard érythémateux inflammatoire, des lésions vésiculo-bulleuses douloureuses à la palpation d'aspect linéaire, strié, dessinant la forme et l'architecture des feuilles et tiges des algues marines, ce placard intéressait toute la région fessière, le reste de l'examen cutanéo-muqueux et somatique étaient sans anomalie. Le diagnostic a été posé cliniquement chez notre patiente. Le traitement reçu était à base de bains abondants; dermocorticoïdes, crème apaisante et antihistaminique. L’évolution était bonne en dehors d'une pigmentation résiduelle.

**Figure 1:**
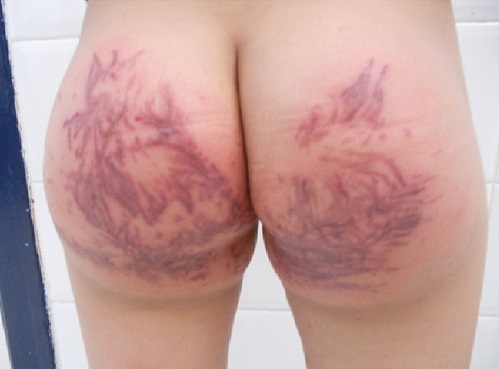
Phytophotodermatose de la région fessiére

